# Attitudes Toward Video Consultations From the Perspective of Physicians and Psychotherapists in German Outpatient Care After the COVID-19 Pandemic: Survey Study

**DOI:** 10.2196/73757

**Published:** 2026-01-06

**Authors:** Lara Kleinschmidt, Juergen Wasem, Nikola Blase, Beatrice Nauendorf, Juliane Malsch, Matthias Brittner, Paul Brandenburg, André Aeustergerling, Theresa Hüer

**Affiliations:** 1 Institute for Health Care Management and Research, University of Duisburg-Essen, Thea-Leymann-Straße 9, Essen, 45127, Germany, 49 2011833774; 2 Kassenärztliche Vereinigung Berlin, Berlin, Germany; 3 Kassenärztliche Vereinigung Westfalen-Lippe, Dortmund, Germany; 4 Kassenärztliche Vereinigung Schleswig-Holstein, Bad Segeberg, Germany; 5 Kassenärztliche Vereinigung Mecklenburg-Vorpommern, Schwerin, Germany

**Keywords:** eHealth, telehealth, telemedicine, remote care, mental health services, provider views, virtual medicine, attitude of health personnel, COVID-19, video consultation, survey, suitable types of treatment, medical fields, German outpatient medical care, physician and psychotherapist perspective, provider perspective

## Abstract

**Background:**

Although video consultations (VCs) are permitted in German outpatient care and have seen a notable rise during the COVID-19 pandemic, their use still does not seem to have become established in Germany.

**Objective:**

This survey aims to evaluate the attitudes of physicians and psychotherapists with regard to the use of VC after the COVID-19 pandemic, in particular in the context of types of treatment and suitable medical fields.

**Methods:**

A standardized questionnaire was sent out to all 34,095 physicians and psychotherapists in 4 German regions. The analysis consisted of both descriptive and inferential statistics. Subgroup analysis included gender, age groups, community size of practice location, VC experience, type and ownership of practice, and area of medical care. Binary logistic regression was conducted to determine whether physicians’ and psychotherapists’ individual factors, organizational factors, or area of medical care were associated with at least monthly VC provision or interest in VC provision.

**Results:**

The response rate was 17.9%, including a total of 5930 participants in the analysis. About 40% (2216/5863) of the physicians and psychotherapists surveyed stated that they offer VC at least once a month. In the area of medical care, the odds ratio (OR) of at least monthly VC provision in psychotherapeutic care was about 8.2 (95% CI 7.4‐1.64; *P*<.001) compared to primary care, whereas in specialist care, the odds for monthly VC provision were approximately 50% lower than in primary care (OR 0.5, 95% CI 0.43-0.59; *P*<.001). Further, female participants have higher odds to provide VC at least once a month (OR 1.163, 95% CI 1.01‐1.34; *P*=.03). The odds for monthly VC provision in older age groups are approximately 60% higher than in the <40 years old age group (OR 0.41, 95% CI 0.32-0.52; *P*<.001). Around 80% (4347/5442) of the participants expressed interest in VC use. The most common occasions for which treatment by VC was reported to be suitable were discussing test results (1422/1896, 75.0%), taking the patient’s medical history (1195/2147, 55.7%), issuing prescriptions for drugs and remedies (793/1204, 65.9%), and the issuing of incapacity certificates for work (677/1042, 65.0%).

**Conclusions:**

There has been an increase in the self-reported uptake of VC among physicians and psychotherapists compared to prepandemic levels, although this remains at a relatively low level in primary and specialist care. A significant proportion of doctors and psychotherapists have expressed an interest in using VC after the pandemic period. However, this self-reported use is not yet reflected in actual usage data, suggesting the need for further investigation into the underlying factors influencing the gap and identifying potential enablers. Further, these self-assessments by service providers on suitable types of treatment and suitable medical fields can inform political decision-making.

## Introduction

The utilization of telemedicine, in particular the implementation of video consultation (VC), has become increasingly popular since the onset of the global COVID-19 pandemic in 2020 [1-3]. In Germany, VC could be billed for certain diagnoses or check-ups in outpatient care since 2017. In 2019, the legislative initiative of the Digital Healthcare Act was implemented with the objective of accelerating the digital transformation of the nation’s health care infrastructure. Thereafter, VCs have been eligible for billing in almost all medical specialties, as physicians and psychotherapists have been granted permission to use them at their discretion, without the prior restrictions [4].

However, uptake in its use was only reported during the COVID-19 pandemic as it was a relevant option for preventing infections [4-6]. Toward the end of the pandemic, its use has declined again and does still not appear to have become established into everyday medical practice [7]. A representative survey of 2800 medical practices in Germany by Albrecht et al [8] revealed that in 2021, 20% of practices offered VC, compared to 25% during the beginning of the COVID-19 pandemic in 2020. Among psychotherapy practices, VCs are used more frequently, with about three quarters of practices offering the service in 2020 and 2021 [8]. Claims data analysis by the statutory health insurance fund BARMER reported that in 2021 around 80% of the VCs have been provided by psychiatrists and psychotherapists, followed by general practitioners (GPs) who provided 14% of all VCs. Less prevalent are the medical specialties gynecologists (1.8%) and pediatricians (1.5%) [7]. According to the latest figures reported by the Zentralinstitut kassenärztliche Versorgung, the use of VC in Germany did not demonstrate an upward trend from 2022 to 2024, with approximately 2.7 million VCs recorded in both years [9].

For the period before and during the COVID-19 pandemic, there are study results that have identified a number of factors that influence the provision of VC services. Besides medical specialty, these include the age of the service provider, the community size of practice location, and the size of the service provider’s practice. A greater uptake in VC use during the COVID-19 pandemic was observed among younger physicians and psychotherapists [6,8] and among providers in an urban practice [6,10]. In terms of practice type, Kane et al [11] and Albrecht et al [8] reported higher use in larger practices, multispecialty practices, or practices with a nonphysician ownership structure. Conversely, Knörr et al [10] found no associations for use but did find associations for perceived benefit with group practices rather than solo practices. At that time, VCs were most frequently employed for the purposes of discussing examination results and taking a patient’s medical history. VCs have previously been reported as being inadequate for the purposes of diagnosis and determining medical indication [8].

Moreover, VCs have been most commonly used as an adjunct to in-person treatment. However, in rural regions with a lower density of physicians, VCs have been increasingly provided without additional personal contact in a billing quarter, that is, in 3 months [6]. This indicates that VC can also be a suitable method in the context of a shortage of medical care. Internationally, service providers have reported positive experiences with chronic disease management, mental health support, and medication management during the pandemic [12]. Despite the fact that experiences with VC during the pandemic have frequently been characterized as positive overall [13], health care providers appear to be rather hesitant about implementing them into routine practice [14].

The sudden and unanticipated need for VC adoption during the course of the pandemic was widely regarded as an opportunity for their long-term integration into routine treatment. At the same time, it accelerated ongoing political initiatives in Northern Europe to digitize health care [15-17]. However, in Germany, measures to promote the use of VC do not seem to have been effectively implemented, which has created uncertainty about the sustainability of VC. This underscores the need for research on how VC use evolved. Although there is plenty of research on providers’ experiences with VC during the pandemic, given the potential shifts in attitudes toward such services resulting thereof, it is crucial to examine the subject from a provider’s perspective and their attitudes toward telemedicine services in a postpandemic context [14]. Hence, the objective of this study is to examine the attitudes of physicians and psychotherapists with regard to the use of VC after the COVID-19 pandemic in German outpatient care. Specifically, the study examines factors associated with VC provision and interest in VC provision. Further, the study investigates which types of treatments and medical fields are deemed suitable by the medical and psychotherapeutic service providers in nonpandemic conditions. This survey of service providers’ perspectives can assist in ascertaining how VC can be used sensibly. For many, their familiarity with VC was built in the context of the pandemic, when it was quickly implemented. There is now a need for evaluation to establish an evidence base for its optimal use [16,18].

## Methods

### Study Design

This survey was conducted as part of the German study “Preference-based use of video consultations in urban and rural regions,” which was funded by the Innovation Fund of the German Federal Joint Committee (funding no. 01VSF20011). A study protocol was published previously [19]. Based on groundwork research within the study—a literature review and focus group discussions—a standardized questionnaire was developed.

The questionnaire was divided into three segments: (1) attitudes and experiences regarding VC provision, (2) preference survey using discrete choice experiments, and (3) sociodemographic information as well as information about the medical profession.

First, the survey asked whether VC was offered and, if so, how frequently. A follow-up question enquired how long VC had been part of their medical treatment. The first section also asked about suitable types of treatment and medical fields for which VC could be (at least partially) suitable. There were also questions about the exclusive use of VC (eg, no additional face-to-face contact in a quarter for the treatment case), the use of VC in treating children and adolescents, and potential future VC provision. All questions had categorical responses except the questions about suitable types of treatment and suitable medical fields for treatment via VC, which were 5-scale Likert questions (highly unsuitable, unsuitable, to some extent, suitable, and very suitable). All Likert scales had an “I don’t know” option, which was coded as missing during the analysis. Likert scales also included open-text response options to allow for further explanation of the participants’ experiences.

The attitude of inhibiting and promoting factors for the provision of VC and the results of the second part of the survey (preference elicitation) are published separately. Thus, no further details are given here.

The third section included sociodemographic questions and information about the participants’ medical profession. These included membership at one of the four participating regional Associations of Statutory Health Insurance Physicians (ASHIPs), community size of practice location, employment status, area of medical care, and medical specialty or psychotherapy practice.

Prior to conducting the survey, a pretest with think-aloud and probing methods was done [20]. The pretest included 6 physicians with different medical specialties.

### Ethical Considerations

At the beginning of the survey, all participants received information about the objective of the study and how their data would be dealt with. Participation was anonymous and voluntary, and participants could opt out at anytime. Entries could be made on paper or online using a QR code using QuestionPro online survey software (QuestionPro, Inc). The incentive to participate in the survey was amplified by the prospect of winning a tablet in a raffle. The local ethics committee of the Medical Faculty of the University of Duisburg-Essen approved the study (reference: 21‐10283-BO). Completion time of the survey was around 20 minutes. An excerpt of the survey can be found in Multimedia Appendix 1.

### Recruitment

The 4 participating ASHIPs in the regions Berlin, Westphalia-Lippe, Mecklenburg-Western Pomerania, and Schleswig-Holstein sent the survey via postal delivery to all their members (physicians and psychotherapists) who met the inclusion criterion. Inclusion criterion was being eligible to provide VC. In Germany, outpatient medical care in the field of psychotherapeutic/psychiatric disorders is provided by medical care providers (eg, medical psychotherapists, psychiatrists, neurologists, family physicians) and nonphysician care providers called psychological psychotherapists. As both groups are allowed to provide VC and are reimbursed under the same remuneration system, psychological psychotherapists were also included in the study. The ASHIPs cover rural as well as urban regions.

In order to gain insight into the postpandemic perspective, the survey was conducted between November 2022 and March 2023. No reminder was sent out, as the target of 10% response rate was reached early.

### Statistical Analysis

Descriptive data are presented as frequencies (n) and percentages (%). All 49 medical specialty categories were aggregated to 12 final medical specialist groups to simplify the evaluation. For the gender, participants who answered “diverse” were coded as missing in the gender variable for the purposes of subgroup analysis. This decision was taken to safeguard the privacy of those involved because of the relatively limited number of participants with this response. As not all questions were mandatory, missing answers were possible, and no further conclusions can be drawn about the number of “diverse” cases.

Subgroup analyses included gender (male or female), age groups (<40 y, 40‐50 y, 51‐60 y, and >60 y), community size in reference to the classification “Stadt-und Gemeindetyp” as rural or urban by the Federal Institute for Research on Building, Urban Affairs, and Spatial Development (rural community, small town, middle town, or large city) [21], VC experience (yes or no), type of practice (individual practice, joint practice, group practice, or medical care unit), ownership of practice (self-employed and employed), and area of medical care (primary care, specialist care, or psychotherapeutic care). Physicians and psychotherapists were categorized as having VC experience if they stated they offered VC at least once a month. In Germany, the distinction between joint practice and group practice is primarily evident in the manner of billing. In joint practice, a joint billing system is employed, whereas in group practice, the physicians share the facilities but operate independently of one another. Medical care units are outpatient facilities in which physicians are employed exclusively and which can be operated by physicians, hospitals, or other licensed providers. With regard to the variable type of practice, multiple responses were permitted in the questionnaire; however, cases with multiple answers were excluded for the subgroup analysis. Regarding areas of medical care, in primary care, all family physicians, including GPs, general internists, and pediatricians, are displayed. Specialist care, on the other hand, comprised physicians who have indicated that they work in specialist care but not in the areas of family medicine and psychiatry or psychotherapy. With regard to psychotherapeutic care, all physicians or nonphysician service providers (psychological psychotherapists) who have reported working in this field were included. All participants working in psychotherapy, psychiatry, or neurology care were referred to in the study as “psychotherapists,” unless otherwise stated.

First, a descriptive analysis was conducted, followed by an association analysis as a second step and binary logistic regression as a third step. Correlation and association coefficients were calculated using the Stuart-Kendall Tau-c for ordinal variables and Pearson *χ*^2^ categorical variables. Effect sizes were indicated by Stuart-Kendall Tau-c for ordinal variables and Cramer *V* for categorical variables [22,23]. In accordance with the criteria established by Cohen (*d*), effect sizes that fall below the threshold of 0 are considered inconsequential and thus excluded from the reporting. Effect sizes greater than 0.1 and up to 0.3 are considered weak, while those greater than 0.3 are considered moderate, and those greater than 0.5 are considered strong [24].

Binary logistic regression was conducted to determine whether physicians’ and psychotherapists’ individual factors (gender, age, and region), organizational factors (ownership or type of practice), or area of medical care are associated with at least monthly VC provision (yes or no). Similarly, it was then examined whether these factors are related to the general interest in VC provision (yes or no). The aim is to test whether the predictors influence the probability of VC provision or interest in VC and to determine their odds. Multicollinearity diagnostics indicated no concerns for the predictor variables (all tolerance values >0.20; all variance inflation factor values <5). All independent variables were included in the analysis simultaneously in order to examine their contribution to predicting each dependent variable. The level of statistical significance was set at *α*=.05, with a *P* value of ≤.05 deemed significant. Statistical analyses were performed using the statistical software IBM SPSS Statistics version 28 (IBM Corp).

## Results

### Response Rate and Participants’ Characteristics

The study questionnaire was sent to 34,095 physicians and psychotherapists, of whom 33,994 participants could be contacted. The remaining 101 questionnaires could not be delivered. The total number of returned questionnaires was 6112, resulting in a response rate of 17.9%. About 60.5% (3700/6112) of participants responded online and 39.5% (2412) responded by paper. Following the exclusion of 182 questionnaires that were either implausible or blank, a total of 5930 respondents were included in the analysis. They represent around 17.4% (5930/34,095) of physicians and psychotherapists in the four regions surveyed.

The study sample comprised a slight overrepresentation of women (3140 to 2378; 56.9% to 32.1%) and a slight underrepresentation of medical service providers in the older age groups (>60 y: 1359/5539, 24.5% to 10,261/34,095, 30.1%) compared to the basic population. Table 1 summarizes the sociodemographic characteristics and information on the medical profession.

**Table 1.** Characteristics and medical profession of survey participants and basic population.

	Participants, n (%)	Physicians and psychotherapists in selected regions, n (%)^a^
Total	5930 (100)	34,095 (100)
Gender (n=5518)		
Female	3140 (56.9)	17,452 (51.2)
Male	2378 (43.1)	16,643 (48.8)
Age groups (aggregated) (n=5539)		
<40 years	749 (13.5)	3538 (10.4)
40‐50 years	1478 (26.7)	8317 (24.4)
51‐60 years	1952 (35.2)	11,979 (35.1)
>60 years	1359 (24.5)	10,261 (30.1)
Community size of practice location (n=5525)		—^b^
Rural community	372 (6.7)	
Small town	965 (17.5)	
Middle town	1411 (25.5)	
Large city	2777 (50.3)	
Employment status^c^ (n=5542)		—
Self-employed	4421 (79.8)	
Employed	1161 (20.9)	
Type of practice^c^ (n=5542)		—
Individual practice	2864 (50.1),	
Joint practice^d^	1526 (26.7)	
Group practice^d^	807 (14.1)	
Medical care unit^d^	521 (9.1)	
Area of medical care (n=5531)		—
Primary care	1591 (28.8)	
Specialist care	1747 (31.6)	
Psychotherapeutic care	2193 (39.6)	

a Data of all 34,095 physicians and psychotherapists from the Associations of Statutory Health Insurance Physicians (ASHIPs) in the regions Berlin, Westphalia-Lippe, Mecklenburg-Western Pomerania, and Schleswig-Holstein. All specialist groups that are theoretically authorized to provide video consultation in Q4 2023 are included. Source: Data provided by the participating ASHIPs.

b Not available.

c Multiple answers possible*.*

d German terms: joint practice: Berufsausübungsgemeinschaft; group practice: Praxisgemeinschaft; medical care unit: Medizinisches Versorgungszentrum.

About half (2777/5525) of the participants have their practice location in a large city, 25.5% (1411/5525) run their practice in a medium-sized town, 17.5% (965/5525) in a small town, and 6.7% (372/5525) in the countryside. Most of the participants (4549/5542, 82.1%) are self-employed. Around half of the participants (2873/5542, 51.8%) run an individual practice, 27.6% (1530/5542) a joint practice, and 14.6% (811/5542) a group practice. Only 9.5% (529/5542) of the participants state that they are employed in a medical care unit. The participants also were asked to indicate their area of medical care. About 28.8% (1591/5531) of the participants practice in primary care, while a comparable proportion (1747/5531, 31.6%) works in specialist care. A larger proportion of participants (2193/5531, 39.6%) is engaged in psychotherapeutic/psychiatric care.

Most common medical care areas are primary care (1438/5484, 26.1%), nonphysician care providers in psychological psychotherapy (1293/5484, 23.6%), physicians in the field of psychotherapy, psychiatry, or neurology (464/5484, 8.5%), and psychotherapists for children and adolescents (458/5484, 8.4%). Other, less frequent areas of medical care in which the participants are trained in include gynecology, gynecological endocrinology, and reproductive medicine (315/5484, 5.7%), orthopedics and trauma surgery (182/5484, 3.3%), pediatrics and adolescent medicine in primary care (153/5484, 2.8%), otorhinolaryngology (130/5484, 2.4%), and venereal diseases and dermatology (113/5484, 2.1%). Table 2 presents a summary of the (aggregated) medical specialties displayed in this survey in comparison to the basic population.

**Table 2.** Medical specialty (aggregated).

	Participants, n (%)	Physicians and psychotherapists in selected regions, n (%)^a^
Total	5484 (100)	34,104^b^ (100)
Primary care (excluding pediatrics in primary care)	1438 (26.1)	10,736 (31.5)
Psychological psychotherapy	1293 (23.6)	5446 (16.0)
Psychotherapy/psychiatry/neurology (excluding psychological psychotherapists and psychotherapists for children/adolescents)	464 (8.5)	2389 (7.0)
Psychotherapists for children and adolescents	458 (8.4)	1590 (4.7)
Gynecology, gynecological endocrinology, and reproductive medicine	315 (5.7)	2394 (7.0)
Orthopedics and trauma surgery	182 (3.3)	1702 (5.0)
Pediatrics (in primary care)	153 (2.8)	1350 (4.0)
Otorhinolaryngology	130 (2.4)	898 (2.6)
Surgery (excluding orthopedics and trauma surgery)	128 (2.3)	863 (2.6)
Venereal diseases and dermatology	113 (2.0)	793 (2.3)
Pediatrics (specialist care)	44 (0.8)	144 (0.4)
Other	766 (13.2)	5799 (17.0)

a Data of all 34,095 physicians and psychotherapists from the Associations of Statutory Health Insurance Physicians (ASHIPs) in the regions Berlin, Westphalia-Lippe, Mecklenburg-Western Pomerania, and Schleswig-Holstein. All specialist groups that are theoretically authorized to provide video consultation in Q4 2023 are included. Source: Data provided by the participating ASHIPs.

b Since in a few cases more than one specialist group was assigned to a physician or psychotherapist in an ASHIP, the number in this table (34,104) differs slightly from the total number of respondents (34,095).

### Provision of Video Consultations

#### Provision of VC

Among the physicians and psychotherapists who have already used VC, 78.3% (1722/2199) have offered them since the beginning of the COVID-19 pandemic; 11.2% (246/2199) started using VC later on during the pandemic (from 2021 onwards). However, only 10.2% (231/2199) have been providing VC since before the COVID-19 pandemic.

Of the physicians and psychotherapists, 37.8% (2216/5863) reported using VC at least once a month, with 36.6% using VC several times a week, 33.6% at least once a week, and 29.5% at least once a month. Meanwhile, 62.2% of participants (3647/5863) report that they have rarely or never offered VC. Significant associations with gender, age, community size of practice location, type of practice, ownership of practice, and area of medical care were identified with VC provision (see Multimedia Appendix 2).

The findings of the logistic regression analysis demonstrate that at least monthly VC provision is associated with each included predictor except community size of practice location. For the area of medical care, the odds ratio (OR) of at least monthly VC provision in psychotherapeutic care was about 8.2 compared to primary care (95% CI 7.4‐1.64; *P*<.001), whereas in specialist care, the odds for monthly VC provision were approximately 50% lower than in primary care (OR 0.5, 95% CI 0.43-0.59; *P*<.001). Regarding medical specialty, within psychotherapeutic care, medical professionals who offer VC most frequently are psychological psychotherapists (985/1282, 76.8%), psychotherapists for children and adolescents (333/456, 73%), and psychotherapists, psychiatrists, and neurologists (245/457, 53.6%). At a considerable distance from the frequency observed in psychotherapeutic care, 22.7% (10/44) of pediatric specialists, 20% (285/1422) of physicians in primary care, and 19.6% (22/112) of specialists for venereal diseases and dermatology have stated to have VC experience.

Individual factors such as gender and age were also significant predictors for monthly VC provision. Female participants have higher odds to provide VC at least once a month (OR 1.163, 95% CI 1.01‐1.34; *P*=.03). The odds for monthly VC provision in older age groups are approximately 60% higher than in the <40 years old age group (OR 0.41, 95% CI 0.32-0.52; *P*<.001). With regard to organizational factors, self-employed participants had about 50% higher odds of providing VC than employed participants (OR 0.480, 95% CI 0.39-0.59; *P*<.001). Regarding the type of practice, participants working in group practice (OR 1.33; 95% CI 1.07‐1.66; *P*=.01) and medical care units (OR 1.39, 95% CI 1.04‐1.85; *P*=.03) had significantly higher odds to provide VC at least once a month compared to those in individual practice. The model was statistically significant, *χ*²_13_=1937.52, *P*<.001, and explained approximately 41% according to Nagelkerke pseudo-*R*². Further, the Hosmer–Lemeshow test was nonsignificant, *χ*²_8_=12.32, *P*=.14, indicating good model fit.

#### Interest in VC Provision

The majority of the physicians and psychotherapists (4347/5442, 79.9%) express interest in potentially providing VC. There appears to be a significant association between interest in use by gender, age group, VC experience, and area of medical care (see Multimedia Appendix 3).

The patterns evident in current at least monthly VC provision are also reflected in the results of the logistic regression analysis of general interest in VC provision. Interest in providing VC is significantly associated with gender, age group, practice location, type of practice, and area of medical care. For instance, logistic regression indicated a significant association between age group and the likelihood of interest in VC provision (*P*<.001). Compared to the reference group of physicians and psychotherapists under 40 years, those aged 40‐50 years had 51% lower odds of the outcome (OR 0.49, 95% CI 0.34-0.69; *P*<.001). The 50‐60 years age group also exhibited lower odds compared to the reference group (OR 0.3, 95% CI 0.21-0.42; *P*<.001). The strongest effect was observed among individuals aged over 60 years, who had approximately 80% lower likelihood of interest in VC provision (OR 0.2, 95% CI 0.15-0.28; *P*<.001). The overall model for interest in VC provision was statistically significant, *χ*²_13_=574.23, *P*<.001, indicating that the set of predictors reliably distinguished interest in use of VC provision. The model explained approximately 17% according to Nagelkerke pseudo-*R*². The Hosmer–Lemeshow goodness-of-fit test was nonsignificant, *χ*²_8_=13.77, *P*=.09, suggesting acceptable model fit. Table 3 presents details on the results of the logistic regression models.

**Table 3.** Binary logistic regression for at least monthly VC^a^ provision and interest in VC provision.

Predictor	At least monthly VC provision^b^ (n=5232)			Interest in VC provision ^c^ (n=5173)		
	*P* value	OR (95% CI)		*P* value	OR (95% CI)	
Gender (reference: male)						
Female	.03	1.163 (1.01-1.34)		.03	1.315 (1.13-1.53)	
Age group (reference: <40 y)						
40‐50 y	<.005	0.711 (0.56-0.90)		<.001	0.486 (0.34-0.69)	
51‐60 y	<.008	0.729 (0.58-0.9)		<.001	0.300 (0.21-0.42)	
>60 y	<.001	0.409 (0.32-0.52)		<.001	0.204 (0.15-0.28)	
Community size of practice location (reference: rural community)						
Small town	.10	0.783 (0.59-1.05)		.79	0.958 (0.7-1.31)	
Middle town	.20	0.833 (0.63-1.10)		.96	0.993 (0.73-1.35)	
Large city	.38	1.128 (0.864-1.47)		.21	1.210 (0.9-1.63)	
Ownership of practice (reference: self-employed)						
Employed	<.001	0.480 (0.39-0.59)		.30	0.893 (0.72-1.11)	
Type of practice (reference: individual practice)						
Joint practice	.05	1.175 (0.999-1.38)		<.001	1.560 (1.31-1.85)	
Group practice	.01	1.331 (1.067-1.66)		<.001	1.385 (1.08-1.78)	
Medical care unit	.03	1.387 (1.039-1.85)		.01	2.713 (1.96-3.76)	
Area of medical care (reference: primary care)						
Specialist care	<.001	0.503 (0.425-0.59)		.01	0.807 (0.68-0.96)	
Psychotherapeutic care	<.001	8.917 (7.472-10.64)		<.001	3.850 (3.11-4.77)	
Constant	0.33	0.845		<.001	5.606	

a VC: video consultation.

b At least monthly VC provision: pseudo-*R*²=0.41.

c Interest in VC provision: pseudo-*R*²=0.17.

### Exclusive Use of VC

Almost half (2702/5651, 47.8%) of the physicians and psychotherapists surveyed can imagine treating patients exclusively via VC without further face-to-face appointments for the treatment case in the same quarter. There are differing attitudes toward the exclusive use of VC, depending on whether the participants have VC experience (*χ*²_1_=320.997, *P*<.001; Cramer *V*=0.239), their age group (*χ*²_3_=126.589, *P*<.001; Cramer *V*=0.152), or their medical specialty (*χ*²_11_=146.092, *P*<.001; Cramer *V*=0.164). Physicians and psychotherapists with VC experience (1345/2676, 63%) are more likely to imagine treating patients in a treatment case exclusively via VC than participants who have not or rarely provided VC before. Further, medical specialists with specialist training in venereal diseases and dermatology (80/113, 70.8%), GPs (720/1423, 50.6%), nonphysician care providers in psychological psychotherapy (721/1285, 56.1%), and physicians who specialized in psychotherapy, psychiatry, and neurology (232/459, 50.5%) are more likely to consider treating patients in a treatment case exclusively via VC.

### Suitable Medical Fields

Regarding suitable medical fields, the participants were asked if parts of their treatment could be carried out via VC for the enquired medical fields. Results only include participants with previous VC experience, as it is assumed that a more precise assessment can be made. The participants indicated a potential for using VC, in particular, in the area of mental and behavioral disorders. In the field of mental disorders, nonorganic sleep disorders (very suitable or suitable: 1296/1995, 65.0%), affective disorders (very suitable or suitable: 1123/1991, 56.4%), anxiety disorders (very suitable or suitable: 1110/2014, 55.1%), sexual dysfunctions (very suitable or suitable: 952/1836, 51.3%), or obsessive-compulsive disorders (very suitable or suitable: 952/1974, 48.2%) are considered to be very suitable or suitable most often. An exception is cases of schizophrenia (highly unsuitable or unsuitable: 1047/1764, 59.4%) as well as schizotypal and delusional disorders caused by psychotropic substances (highly unsuitable or unsuitable: 824/1837, 44.9%), which are rather reported as highly unsuitable or suitable for VC.

Among chronic illnesses, the treatment of chronic pain (very suitable or suitable: 936/1517, 61.7%), metabolic disorders (very suitable or suitable: 464/1009, 46.0%), and allergies (very suitable or suitable: 413/1020, 40.5%) as well as dermatological diseases (very suitable or suitable: 368/989, 37.2%) are considered very suitable or suitable for VC treatment.

Acute illnesses are rather considered highly unsuitable or unsuitable, with the exception of acute headaches (to some extent suitable: 481/1238, 38.9%) or dermatoses (to some extent suitable: 337/922, 36.6%), which are considered suitable to some extent. Further details on the attitude of suitable medical fields from the perspective of physicians and psychiatrists with VC experience can be found in Multimedia Appendix 4.

### Types of Treatment

To assess potential suitable types of treatment, only findings of participants with VC experience are displayed as a better understanding is assumed. Significant associations have been identified by participants who have experience in VC provision for all queried types of treatment. They are more likely to consider the types of treatment as suitable for VC provision than participants with little or no experience; for details on their associations and effect sizes, see Multimedia Appendix 5.

According to over 60% of these physicians and psychotherapists, discussing test results (1422/1896, 75%), issuing of prescriptions for drugs and remedies (793/1204, 65.9%), and issuing of incapacity certificates (677/1042, 65.0%) and treatment planning (1379/2131, 64.7%) are very suitable or suitable types of treatment via VC (see Table 4). Follow-up appointments (771/1350, 57.1%) and taking a patient’s medical history (1195/2147, 55.7%) are considered very suitable or suitable by over half of the participants. Diagnostic procedures appear to be the least suitable form of treatment for VC, with only 24.6% of participants (very suitable or suitable: 523/2126), indicating agreement with these types of treatment.

**Table 4.** Attitudes of physicians and psychotherapists with VC^a^ experience towards the suitability of types of treatment for VC.

	Highly unsuitable, n (%)	Unsuitable, n (%)	To some extent, n (%)	Suitable, n (%)	Very suitable, n (%)
Discussion of test results (n=1896)	29 (1.5)	101 (5.3)	344 (18.1)	706 (37.2)	716 (37.8)
Medical history (n=2147)	85 (4.0)	343 (16.0)	524 (24.4)	633 (29.5)	562 (26.2)
Issuing prescriptions for drugs and remedies (n=1204)	49 (4.1)	136 (11.3)	226 (18.8)	429 (35.6)	364 (30.2)
Issuing incapacity certificate (n=1042)	44 (4.2)	100 (9.6)	221 (21.2)	366 (35.1)	311 (29.8)
Therapy treatment planning (n=2131)	35 (1.6)	172 (8.1)	545 (25.6)	919 (43.1)	460 (21.6)
Individual psychiatric/psychotherapeutic consultations (n=2017)	27 (1.3)	122 (6.0)	546 (27.1)	743 (36.8)	579 (28.7)
Follow-up checks (eg, wound healing, medication) (n=1350)	34 (2.5)	130 (9.6)	415 (30.7)	485 (35.9)	286 (21.2)
Group sessions (eg, in psychotherapy) (n=977)	178 (18.2)	381 (39.0)	256 (26.2)	105 (10.7)	57 (5.8)
(Further) diagnostic work-up (n=2126)	151 (7.1)	669 (31.5)	783 (36.8)	375 (17.6)	148 (7.0)

a VC: video consultation.

Regarding area of medical care of participants with VC experience, significant associations have been identified for taking a patient’s medical history (*χ*²_8_=21.266, *P*<.001; Cramer *V*=0.229), further diagnostic work-up (*χ*²_8_=85.333, *P*<.001; Cramer *V*=0.145), issuing an incapacity certificate (*χ*²_8_=36.051, *P*<.001; Cramer *V*=0.135), the discussion of test results (*χ*²_8_=164.154, *P*<.001; Cramer *V*=0.213), follow-up checks (*χ*²_8_=71.743, *P*<.001; Cramer *V*=0.167), and individual psychiatric or psychotherapeutic consultations (*χ*²_8_=127.499, *P*<.001; Cramer *V*=0.182). Taking a patient’s medical history via VC is deemed very suitable or suitable rather by providers in primary care (very suitable or suitable: 259/308, 84.1%) and specialist care (very suitable or suitable: 141/198, 71.2%), less than by providers in psychotherapeutic care (very suitable or suitable: 739/1544, 47.9%). It is similar in the case of discussing test results, 91.2% (281/308) of GPs and 90.2% (175/194) of specialists report them to be a very suitable or suitable option via VC, while 68.6% (895/1305) of psychotherapists consider them very suitable or suitable. Conversely, the attitudes of the suitability of (further) diagnostics are the opposite. Although (further) diagnostics are deemed highly unsuitable or suitable, health care professionals engaged in the delivery of psychotherapeutic care (very suitable or suitable: 441/1526, 28.9%) are more likely to view them as suitable than GPs (very suitable or suitable: 37/303, 12.2%) and specialists (very suitable or suitable: 19/201, 9.5%). For follow-up checks, 64.5% (517/802) of psychotherapists and about half of specialists (98/184, 53.3%) consider these very suitable or suitable for VC, whereas only 38.3% (116/303) of GPs agree on their suitability. In particular, in the context of individual psychotherapeutic consultations, psychotherapists (very suitable or suitable: 1079/1569, 68.8%) consider these to be suitable for VC, and also approximately 50% of GPs (very suitable or suitable: 122/241, 50.6%) and specialist care (very suitable or suitable: 55/111, 49.5%) hold this view. For further associations or correlations and effect sizes, see Multimedia Appendix 6.

## Discussion

### Principal Findings

This study examined the attitude toward the use of VC in German outpatient care after the COVID-19 pandemic from a provider perspective. For the majority of the surveyed physicians and psychotherapists, the advent of the COVID-19 pandemic served as a catalyst, with approximately 80% commencing VC use during the initial phase of the pandemic since 2020, particularly within the area of psychotherapeutic care. VC provision is reported more often in psychotherapeutic care (73%) and still less often in primary (20%) and specialist care (12%). Despite over half of service providers reporting little or no VC provision, with approximately 40% stating otherwise, this represents a notable increase in providers who have used VC so far compared to the prepandemic period. However, the survey results do not reveal whether they have incorporated VC into their daily routine or whether it is merely a sporadic occurrence necessitated by the circumstances during the pandemic. Current claims data analyses indicate that VC provision is not regular, as use has experienced a decline once again following the conclusion of the COVID-19 pandemic [7].

According to our findings, the odds of VC provision are higher among female physicians and psychotherapists, younger age groups, and those living in urban areas. These results are coherent with previously published research on German claims data done earlier in the study by Hüer et al [6] covering the time period during the COVID-19 pandemic. Therefore, there seem to be no changes in the analyzed factors associated with VC provision during and after the COVID-19 pandemic. In terms of organizational factors, practice types with shared facilities demonstrated higher odds for VC provision than participants in an individual practice setting. During the pandemic, Hüer et al [6] and Knörr et al [10] did not observe a higher frequency of use by group practices. However, Knörr et al [10] noted that the perceived benefit is greater in group practices. Recent systematic reviews emphasized the importance of perceived usefulness and ease of use in the adoption of telemedicine [25,26]. The findings of this study suggest that shared facilities may result in a reduction of organizational barriers to VC use and offer enhanced practical benefits or ease of use.

Conversely, self-employed physicians also demonstrated higher odds for VC provision. The current literature suggests that the barriers are particularly related to the expected usefulness within their professional environment, making the indications inconclusive.

Globally, many countries have integrated telehealth into routine care to some extent, although the degree varies widely [27]. The 2022 International Health Policy Survey of General Practitioners, conducted by the Commonwealth Fund in 10 high-income countries, examined opinions on the user-friendliness and effectiveness of telemedicine in the period following the peak of the pandemic. In the majority of countries, physicians reported a number of advantages, including financial compensation, improved timeliness of care, and enhanced ability for mental health care. German physicians demonstrated the lowest levels of satisfaction across the majority of stated metrics, while medical professionals in the United Kingdom, Australia, Canada, and the United States reported higher levels of satisfaction and ease of implementation of the telehealth platform. In Germany, telehealth expanded more slowly postpandemic, with only about 30%‐40% of German primary care doctors stating telehealth to be easy to implement, citing cost of the platform as a barrier [28]. This indicates that besides readiness of users and providers, national policies and technical infrastructure have an influence on VC adoption [16,28]. Nevertheless, the results suggest that there is potential to establish use among medical service providers, as approximately 80% expressed interest in potentially using VC. With younger providers in particular using and being interested in using VC, it is likely that an age-related dip in VC provision will diminish in the future. In addition, these results can also help identify subgroups that might perceive more barriers in VC adoption. Elder and male physicians and psychotherapists showed less odds of VC adoption. Research suggests that those groups show less readiness and might lack familiarity or trust in new technologies [26,29]. For the time being, it is recommended that efforts are made to facilitate use [26], with a particular focus on engaging older age groups, as even around 70% of physicians and psychotherapists with little or no VC experience reported interest in using VC. This objective could be realized through the implementation of knowledge pertaining to the advantages of VC in the obligatory Continuing Medical Education administered by the German Medical Associations.

Although awareness of VC has been heightened by the COVID-19 pandemic and there is considerable interest in its use, VC still appears to be used irregularly at present [7], suggesting that there may currently be inhibiting factors inhibiting the expansion of VC provision (eg, concerns about a deterioration in the physician–patient relationship [13] or factors relating to the health care system [16]). This issue merits a need for further research to investigate the reasons for the current reluctance to use VC. In order to explore why the uptake of VC has not been sustainable and to develop strategies that can be used to ensure its long-term integration, a look at hindering factors, particularly in the German health care system, or qualitative research on sustainable implementation strategies would also be an appropriate approach.

Globally, many countries have integrated telehealth into routine care to some extent, although the degree varies widely [27]. The 2022 International Health Policy Survey of General Practitioners, conducted by the Commonwealth Fund in 10 high-income countries, examined opinions on the user-friendliness and effectiveness of telemedicine in the period following the peak of the pandemic. In the majority of countries, physicians reported a number of advantages, including financial compensation, improved timeliness of care, and enhanced ability for mental health care. German physicians demonstrated the lowest levels of satisfaction across the majority of stated metrics, while medical professionals in the United Kingdom, Australia, Canada, and the United States reported higher levels of satisfaction and ease of implementation of the telehealth platform. In Germany, telehealth expanded more slowly postpandemic, with only about 30%‐40% of German primary care doctors stating telehealth to be easy to implement, citing cost of the platform as a barrier [28]. This indicates that besides readiness of users and providers, national policies and technical infrastructure have an influence on VC adoption [16,28]. Nevertheless, the results suggest that there is potential to establish use among medical service providers, as approximately 80% expressed interest in potentially using VC. With younger providers in particular using and being interested in using VC, it is likely that an age-related dip in VC provision will diminish in the future. In addition, these results can also help identify subgroups that might perceive more barriers in VC adoption. Elder and male physicians and psychotherapists showed less odds of VC adoption. Research suggests that those groups show less readiness and might lack familiarity or trust in new technologies [26,29]. For the time being, it is recommended that efforts are made to facilitate use [26], with a particular focus on engaging older age groups, as even around 70% of physicians and psychotherapists with little or no VC experience reported interest in using VC. This objective could be realized through the implementation of knowledge pertaining to the advantages of VC in the obligatory Continuing Medical Education administered by the German Medical Associations. In light of the heightened awareness of VC brought on by the COVID-19 pandemic and given the great interest in the use of VC, VCs still seem to be used irregularly [7], which suggests that there might currently be inhibiting factors that impede the expansion of VC provision (eg, fear of deterioration in the physician–patient relationship [13] or health system factors [16]). This issue merits a need for further research to investigate the reasons for the current reluctance to use VC. In order to explore why the uptake of VC has not been sustainable and to develop strategies that can be used to ensure its long-term integration, a look at hindering factors, particularly in the German health care system, or qualitative research on sustainable implementation strategies would also be an appropriate approach.

Regarding VC as an add-on service or as a service without face-to-face patient contact in the same quarter for the treatment case, there is no clear agreement or rejection in favor of its exclusive use. According to the findings of the study, a slightly below-average number of respondents were in favor of exclusive use. However, it should be noted that in Germany, discounts in honorarium are applied when VC is used exclusively during a given billing quarter [4]. Hence, the attitude of suitability of exclusive VC use may be biased due to the German billing regulations. Nevertheless, the findings suggest that exclusive use may be more appropriate for certain medical specialties. Physicians in venereal diseases and dermatology, as well as nonphysician care providers in psychological psychotherapy, demonstrated a greater tendency to consider the exclusive use of VC.

This research suggests that VC may be particularly suited to the care of patients with mental and behavioral health problems [30]. However, there are limitations for certain diagnoses, such as schizophrenia, schizotypal, and delusional disorders caused by psychotropic substances. This assessment has been widely acknowledged in relevant literature and is accompanied by an increase in the use of VC and perceived suitability by providers of psychotherapeutic care [31,32]. Furthermore, the results suggest that VCs appear to be suitable for selected chronic illnesses. Current research also validates this for selected indications, for example, for diabetes [33], obstructive sleep apnea [34], or dermatological diseases such as atopic dermatitis [35].

Regarding the findings on types of treatment, there are indications that physicians and psychotherapists consider VC to be particularly suitable to those types of treatment which are mainly based on conversations (eg, discussing test results or taking the patient’s medical history, therapy treatment planning) or which require a higher degree of administrative work (eg, issuing of prescriptions for drugs and remedies and issuing of incapacity certificates). This may particularly apply to well-known patients and those with long-term conditions [12,13]. The results of our study, as well as existing research, indicate that diagnostics may not be the optimal fit for this particular modality of treatment in primary and specialist care. For psychotherapeutic care, further diagnostic workup has been rated more positively, which is supported by another German survey [8]. During the COVID-19 pandemic, follow-up checks were reported to be a well-accepted type of treatment [12,36,37]. According to this study, after the COVID-19 pandemic, this was not fully confirmed, with only 33% finding it suitable and 36% suitable to some extent. The variability of the suitability of follow-up checks seems to be dependent on different treatments. In the area of psychotherapeutic care, follow-up visits are more likely to be considered appropriate than in primary care or specialist care. Moreover, differences are observed with regard to the presence or absence of VC experience. This suggests that as a result of the adoption of VC by physicians and psychotherapists, attitudes have shifted toward a more positive outlook. It can be reasonably concluded that training programs may assist in reducing the current deficit of knowledge and in fostering greater confidence in the provision of VC.

### Limitations

The study achieves a satisfactory response rate of around 18% and thus results in a large sample size of 5930, which is typically challenging to obtain when studying physicians or psychotherapists. This was made possible due to the involvement of ASHIPs in contacting all of the physicians in their respective regions. However, it is important to note that the participants do not fully represent the original population. The sample comprises a slightly higher proportion of female participants and a slightly higher proportion of individuals under the age of 40 than is observed in the basic population. The mode of data collection did not influence the variables related to the main findings. However, differences were observed in demographic characteristics such as the age subgroup between participants recruited online and those surveyed via paper-pencil. The option to participate on paper next to online via a QR code was incorporated with the objective of mitigating the exclusion of offline participants, a demographic which is predominantly composed of older individuals. Moreover, it should be acknowledged that there is a considerably greater number of physicians and medical service providers from the psychotherapeutic care area than represented in the surveyed basic population. This could potentially introduce a selection bias, as psychotherapists are more likely to use VC and may therefore have responded differently from the average due to their vested interest in VC. Psychotherapists may perceive VC as being congruent with their professional practice, particularly with regard to continuity of care and emergency situations [1,2,30,38]. Conversely, some medical practitioners, particularly those specializing in physical disciplines, may regard VC as being incongruent with the necessity for physical examinations [39]. Subsequently, the results may lead to more positive attitudes toward VC, which could restrict the generalizability of the findings to other outpatient providers. Subgroup analysis differentiated according to the areas of primary care, and specialist and psychotherapeutic cares was done in order to be able to make a more precise distinction.

While the rollout of VCs has been comprehensively researched in many countries since the start of the pandemic, there has been limited investigation into this for the German outpatient sector up to now. This study, therefore, adds value by taking a closer and more detailed look at the situation in outpatient care in Germany. Yet, physicians and psychiatrists, as well as psychotherapists working exclusively in the private sector, were not included. However, as approximately 90% of insured persons in Germany have statutory health insurance and only members of ASHIPs are permitted to bill patients with statutory health insurance, the relevance of exclusively private practitioners is negligible. Nevertheless, since only German physicians and psychotherapists were surveyed, the transferability of the results to other countries with different regulatory systems may be limited. Further, studies could explore the use of VC in the inpatient or care settings where there may also be potential [40,41]. Additionally, all specific medical specialties in the field of psychiatric/neurological and psychotherapeutic care are referred to as “psychotherapists” in this study. It is important to note that there are differences in practicing depending on specific medical specialty. Further research could go into depth according to specific medical specialties.

While the logistic regression models provided useful insights, several limitations should be noted. First, as this was a cross-sectional study, the associations identified cannot be interpreted causally. The logistic regression model was employed for the purpose of exploratory analysis, with the objective of ascertaining which variables exert a significant effect when controlling for the other variables. Second, although the first model showed substantial explanatory power (Nagelkerke *R*²=0.41) and the second model moderate power (Nagelkerke *R*²=0.17), a certain proportion of variance remains unexplained, suggesting that additional factors not included in the analysis may influence the outcome. These might include digital maturity, technical infrastructure, and remuneration terms [42-44].

As this was an exploratory cross-sectional study, a self-constructed questionnaire was used. Although no full psychometric validation was conducted, the instrument was informed by prior qualitative analyses and pretesting to ensure content relevance and comprehensibility. This limits the extent to which the results can be generalized, and the approach is appropriate for an exploratory design and provides valuable initial insights to guide future research. Furthermore, systematic errors cannot be completely excluded. In this study, the use of batteries of items (eg, Likert scales) may have led to a tendency for the questions to be averaged or not to be differentiated. Concerning measures of association or correlation, the calculated effect sizes are according to Cohen value categorized as weak and a few as moderate.

### Conclusion

Although the actual use of VC in general practice and specialist care is still relatively low, most physicians and psychotherapists are in favor of their use after the COVID-19 pandemic. A wide range of medical indications is considered to be suitable, particularly in the area of psychotherapeutic care and use for chronic illnesses. The findings indicate that German service providers do not appear to be averse to the use of VC as they show high interest in their provision. Potential barriers to the exclusive use of VC may include factors such as remuneration or the medical specialty of the practitioner. Furthermore, in the case of certain medical specialties, treatment, particularly diagnosis, cannot be provided without a physical examination. It is advisable to incorporate the providers’ perspectives into the ongoing refinement of VC policies and practices, as these insights are important for ensuring the successful implementation of VC as a therapeutic modality. This research on physicians’ and psychotherapists’ attitudes adds to a baseline knowledge of different attitudes by providers and can provide grounds to create a shared vision, which has shown to be a driver in the adoption process of digital technology [45]. Besides, the attitude and conduct of medical professionals play a fundamental role in ensuring that patients experience a sufficient level of comfort during the course of their treatment [46]. Given that physicians and psychotherapists are more likely to endorse video-based treatment modalities when they have prior experience with them, expanding training programs may help reduce knowledge gaps, alleviate uncertainty, and foster greater confidence in their use in outpatient care. The expansion of the digital infrastructure in the future, which facilitates the dissemination of information, has the potential to diminish potential barriers. Nevertheless, the results do not provide insights into the hurdles that exist in daily use and how exactly their use can be promoted. Further research is necessary to ascertain the full implications.

## Supplementary material

10.2196/73757 Multimedia Appendix 1 Excerpt of the survey.

10.2196/73757 Multimedia Appendix 2 Subgroup analysis for provision of video consultations.

10.2196/73757 Multimedia Appendix 3 Subgroup analysis for interest in video consultation provision.

10.2196/73757 Multimedia Appendix 4 Attitudes on suitable medical fields (only for participants with video consultation experience).

10.2196/73757 Multimedia Appendix 5 Association and effect size of suitable types of treatment for participants with video consultation experience.

10.2196/73757 Multimedia Appendix 6 Association/correlation and effect size of suitable types of treatment (only for participants with video consultation experience).

## Abbreviations

ASHIP: Association of Statutory Health Insurance Physicians
GP: general practitioner
OR: odds ratio
VC: video consultation
