# Supplementary material for: Attitudes Toward Video Consultations From the Perspective of Physicians and Psychotherapists in German Outpatient Care After the COVID-19 Pandemic: Survey Study
Source: J Med Internet Res. 2026 Jan 6;28:e73757. doi: 10.2196/73757 (PMC12774393; doi:10.2196/73757)
Supplement: Multimedia Appendix 1 [file jmir-v28-e73757-s001.docx]

## **Appendix 1: Excerpt of the survey**

**Erfahrungen und Einstellungen zur Videosprechstunde**

1. **Haben Sie bei Ihrer Kassenärztlichen Vereinigung angezeigt, Videosprechstunden mit einem zertifizierten Videodienstanbieter nutzen zu wollen?**

|  | ja | 🡪 weiter mit **Frage 2** |
| --- | --- | --- |
|  | Das ist bei der KV Mecklenburg-Vorpommern nicht erforderlich. |  |
|  | nein | 🡪 weiter mit **Frage 4** |

1. **Wie häufig setzen Sie Videosprechstunden ein?**

|  | mehrfach in der Woche | 🡪 weiter mit **Frage 3** |
| --- | --- | --- |
|  | mindestens einmal in der Woche |  |
|  | mindestens einmal im Monat |  |
|  | seltener als einmal im Monat | 🡪 weiter mit **Frage 4** |
|  | noch nie |  |

1. **Seit wann setzen Sie Videosprechstunden in der ärztlichen Versorgung ein?**

|  | bereits vor Beginn der COVID-19-Pandemie | 🡪 weiter mit **Frage 5** |
| --- | --- | --- |
|  | seit Beginn der COVID-19-Pandemie  (ab 2020) |  |
|  | erst im späteren Verlauf der  COVID-19-Pandemie (ab 2021 oder später) |  |

1. **Was hat Sie bisher daran gehindert, Videosprechstunden zu nutzen?**

**[Bitte setzen Sie in jeder Zeile genau ein Kreuz.]**

|  | **trifft gar nicht zu** | **trifft eher nicht zu** | **trifft**  **eher zu** | **trifft voll zu** | **keine Angabe** |  |
| --- | --- | --- | --- | --- | --- | --- |
| **Meine** **Internetverbindung** ist nicht gut genug. |  |  |  |  |  |  |
| Die **Internetverbindung meiner**  **Patient/-innen** ist oft nicht gut genug. |  |  |  |  |  |  |
| Mir fehlt die **technische Ausstattung** (z.B. Kamera). |  |  |  |  |  |  |
| Mir fehlt die **Erfahrung mit der**  **technischen Umsetzung**. |  |  |  |  |  |  |
| Ich befürchte, dass die **Beziehung zu meinen Patient/-innen** darunter leidet. |  |  |  |  |  |  |
| Ich befürchte, dass die **Versorgungsqualität** darunter leidet. |  |  |  |  |  |  |
| Ich habe **Bedenken beim Datenschutz**. |  |  |  |  |  |  |
| Ich befürchte einen **Anstieg unnötiger Inanspruchnahmen**. |  |  |  |  |  |  |
| Ich habe **keine Zeit**, neben meinem  regulären Praxisbetrieb Videosprechstunden anzubieten. |  |  |  |  |  |  |
| Ich bin mit der **Vergütung** von Videosprechstunden **nicht zufrieden**. |  |  |  |  |  |  |
| Ich finde die Abrechnungsbegrenzungen für die Videosprechstunde durch die **Quotierungsregelungen** störend. |  |  |  |  |  |  |
| Die **organisatorischen und rechtlichen Aufwände** sind mir zu hoch. |  |  |  |  |  |  |
| Ich finde Videosprechstunden **zu**  **anstrengend**. |  |  |  |  |  |  |
| Meine **Patient/-innen wünschen keine Videosprechstunden.** |  |  |  |  |  |  |
| sonstiger Grund:  [Bitte im Feld rechts eintragen.] |  | | | | | |

1. **Wie gut ist die Videosprechstunde Ihrer Meinung nach für die folgenden Versorgungsanlässe geeignet?**

[Hinweis: Bei dieser Frage geht es um Ihre Meinung und nicht um eine allgemeingültige Aussage, die auf jeden Einzelfall zutrifft.]

**[Bitte setzen Sie in jeder Zeile genau ein Kreuz.]**

|  | **absolut ungeeignet** | **nicht gut geeignet** | **teils, teils** | **gut geeignet** | **sehr gut geeignet** | **keine Angabe** |
| --- | --- | --- | --- | --- | --- | --- |
| Anamnese |  |  |  |  |  |  |
| (weitergehende) Diagnostik |  |  |  |  |  |  |
| Therapieplanung |  |  |  |  |  |  |
| Ausstellung von Rezepten/ Verordnungen |  |  |  |  |  |  |
| Ausstellung von  AU-Bescheinigungen |  |  |  |  |  |  |
| Besprechung von  Untersuchungsergebnissen |  |  |  |  |  |  |
| Verlaufskontrollen (z.B. Wundheilung, Medikation) |  |  |  |  |  |  |
| Psychiatrische/psychotherapeutische Einzelgespräche |  |  |  |  |  |  |
| Gruppensitzungen  (z.B. in der Psychotherapie) |  |  |  |  |  |  |
| sonstiger Anlass:  [Bitte im Feld rechts eintragen.] |  | | | | | |

1. **Könnten Sie sich bei diesen Indikationen vorstellen, dass Teile der ärztlichen Versorgung auch in Videosprechstunden erbracht werden können?**

**[Bitte setzen Sie in jeder Zeile genau ein Kreuz.]**

|  | **absolut ungeeignet** | **nicht gut**  **geeignet** | **teils, teils** | **gut**  **geeignet** | **sehr gut**  **geeignet** | **keine Angabe** |
| --- | --- | --- | --- | --- | --- | --- |
| 1. **chronische bzw. intermittierend auftretende Erkrankungen** | | | | | | |
| Chronische Schmerzen,  z.B. Kopfschmerzen |  |  |  |  |  |  |
| Herz-Kreislauf-Erkrankungen,  z.B. arterielle Hypertonie, KHK |  |  |  |  |  |  |
| Gefäßerkrankungen, z.B. pAVK |  |  |  |  |  |  |
| Lungenerkrankungen,  z.B. Asthma bronchiale, COPD |  |  |  |  |  |  |
| Magen-Darm-Erkrankungen,  z.B. chron. entz. Darmerkrankungen |  |  |  |  |  |  |
| Stoffwechselerkrankungen,  z.B. Diabetes mellitus |  |  |  |  |  |  |
| Augenerkrankungen, z.B. Katarakt |  |  |  |  |  |  |
| HNO-Erkrankungen,  z.B. chronische Rhinosinusitis |  |  |  |  |  |  |
| Neurologische Erkrankungen,  z.B. Zustand nach Schlaganfall |  |  |  |  |  |  |
| Erkrankungen des Muskel-Skelett-Systems, z.B. Rückenschmerzen |  |  |  |  |  |  |
| Dermatologische Erkrankungen,  z.B. Psoriasis |  |  |  |  |  |  |
| Allergien |  |  |  |  |  |  |
| Tumorerkrankungen |  |  |  |  |  |  |
| 1. **akute Erkrankungen** | | | | | | |
| Entzündungen am Auge |  |  |  |  |  |  |
| Entzündungen am Ohr |  |  |  |  |  |  |
| Infektionen der Atemwege |  |  |  |  |  |  |
| Infektionen des Magen-Darm-Traktes |  |  |  |  |  |  |
| Infektionen der Harnorgane |  |  |  |  |  |  |
| Kopfschmerzen |  |  |  |  |  |  |
|  | **absolut ungeeignet** | **nicht gut**  **geeignet** | **teils, teils** | **gut**  **geeignet** | **sehr gut**  **geeignet** | **keine Angabe** |
| Schmerzen im Muskel-Skelett-System |  |  |  |  |  |  |
| Hautverletzungen |  |  |  |  |  |  |
| Dermatosen |  |  |  |  |  |  |
| Geschlechtserkrankungen |  |  |  |  |  |  |
| 1. **Psychische und Verhaltensstörungen** | | | | | | |
| Affektive Störungen (F30-F39) |  |  |  |  |  |  |
| Angststörungen (F40, F41) |  |  |  |  |  |  |
| Zwangsstörungen (F42) |  |  |  |  |  |  |
| Somatoforme Störungen (F45) und Dissoziative Störungen (F44) (Konversionsstörungen) |  |  |  |  |  |  |
| Reaktionen auf schwere Belastungen und Anpassungsstörungen (F43) |  |  |  |  |  |  |
| Essstörungen (F50) |  |  |  |  |  |  |
| Nichtorganische Schlafstörungen (F51) |  |  |  |  |  |  |
| Sexuelle Funktionsstörungen (F52) |  |  |  |  |  |  |
| Persönlichkeits- und Verhaltensstörungen (F60-F69) |  |  |  |  |  |  |
| Verhaltens- und emotionale Störungen mit Beginn in der Kindheit und Jugend (F90-F98) |  |  |  |  |  |  |
| Schizophrenie, schizotype und wahnhafte Störungen (F20-F29) |  |  |  |  |  |  |
| Psychische und Verhaltensstörungen durch psychotrope Substanzen (F10-19) |  |  |  |  |  |  |

1. **Halten Sie es für sinnvoll, Videosprechstunden mit Kindern und Jugendlichen durchzuführen?**

|  | ja, mit Kindern (bis 13 Jahre) und mit Jugendlichen (ab 14 Jahre) |
| --- | --- |
|  | ja, aber nur mit Jugendlichen (ab 14 Jahre) |
|  | nein, weder mit Kindern noch mit Jugendlichen |

1. **Können Sie sich grundsätzlich vorstellen, einzelne Patient/-innen in einem Behandlungsfall auch ausschließlich in Videosprechstunden zu behandeln?**

|  | ja |
| --- | --- |
|  | nein |

1. **Was wäre Ihnen wichtig, um Videosprechstunden gut einsetzen zu können?**

**[Bitte setzen Sie in jeder Zeile genau ein Kreuz.]**

|  | **stimme nicht zu** | **stimme**  **eher nicht zu** | **stimme eher zu** | **stimme voll zu** | **keine Angabe** |
| --- | --- | --- | --- | --- | --- |
| **Schulungsangebote** zur Einführung **für mich und mein Praxisteam** |  |  |  |  |  |
| **Schulungsangebote für meine**  **Patient/-innen** |  |  |  |  |  |
| **einfache/intuitive Bedienung**  der Video-Software |  |  |  |  |  |
| **Video-Software** mit **vielen Funktionen** (z.B. Chat, Einbindung der ePA) |  |  |  |  |  |
| **Video-Software** funktioniert stabil (ohne „Ruckeln“) |  |  |  |  |  |
| angepasste **Vergütungsstruktur und**  **-höhe** (z.B. keine/angepasste Abschläge bei ausschließlicher Nutzung, Quotierung) |  |  |  |  |  |
| hohes **Datenschutzniveau** |  |  |  |  |  |
| klare/transparente **Haftungsregelungen** |  |  |  |  |  |
| **Unterstützung in der Kommunikation mit Patient/-innen anderer**  **Muttersprache** (z.B. Untertitel) |  |  |  |  |  |
| Sonstiges:  [Bitte im Feld rechts eintragen.] |  | | | | |

**Angaben zu Ihrer Person**

1. **Welches Geschlecht haben Sie?**

|  | männlich |
| --- | --- |
|  | weiblich |
|  | divers |

1. **Welcher Altersgruppe gehören Sie an?**

|  | bis 40 Jahre |
| --- | --- |
|  | 41 bis 50 Jahre |
|  | 51 bis 60 Jahre |
|  | über 60 Jahre |

1. **Welcher Kassenärztlichen Vereinigung (KV) gehören Sie an?**

|  | KV Berlin |
| --- | --- |
|  | KV Mecklenburg-Vorpommern |
|  | KV Schleswig-Holstein |
|  | KV Westfalen-Lippe |

1. **Wie würden Sie den Standort Ihrer Praxis beschreiben?**

[Hinweis: „In der Nähe“ meint, dass die Großstadt (mindestens 100.000 Einwohner) in ca. 30 Minuten mit dem Auto erreichbar ist.]

|  | Landgemeinde (weniger als 5.000 Einwohner **mit** Großstadt in der Nähe) |
| --- | --- |
|  | Landgemeinde (weniger als 5.000 Einwohner **ohne** Großstadt in der Nähe) |
|  | Kleinstadt (5.000 bis 20.000 Einwohner **mit** Großstadt in der Nähe) |
|  | Kleinstadt (5.000 bis 20.000 Einwohner **ohne** Großstadt in der Nähe) |
|  | Mittelstadt (über 20.000 bis 100.000 Einwohner **mit** Großstadt in der Nähe) |
|  | Mittelstadt (über 20.000 bis 100.000 Einwohner **ohne** Großstadt in der Nähe) |
|  | Großstadt (mehr als 100.000 Einwohner) |

1. **Ich arbeite …**

[Mehrfachantworten sind möglich.]

|  | selbständig in einer Einzelpraxis. |
| --- | --- |
|  | angestellt in einer Einzelpraxis. |
|  | selbständig in einer Berufsausübungsgemeinschaft (vormals Gemeinschaftspraxis). |
|  | angestellt in einer Berufsausübungsgemeinschaft (vormals Gemeinschaftspraxis). |
|  | selbständig in einer Praxisgemeinschaft. |
|  | angestellt in einer Praxisgemeinschaft. |
|  | selbständig in einem MVZ. |
|  | angestellt in einem MVZ. |

1. **In welchem Versorgungsbereich sind Sie tätig?**

|  | hausärztliche Versorgung |
| --- | --- |
|  | fachärztliche Versorgung |

1. **Welche (fachärztliche) Weiterbildung haben Sie abgeschlossen?**

[Mehrfachantworten sind möglich.]

|  | Allgemeinmedizin |  | Kinderchirurgie |
| --- | --- | --- | --- |
|  | Anästhesiologie |  | Kinder-Hämatologie und -Onkologie |
|  | Angiologie |  | Kinder-Kardiologie |
|  | Augenheilkunde |  | Kinder-Pneumologie |
|  | Chirurgie |  | Mund-Kiefer-Gesichtschirurgie |
|  | Chirurgie/Rheumatologie |  | Nephrologie |
|  | Endokrinologie und  Diabetologie |  | Nervenheilkunde/Neurologie und  Psychiatrie |
|  | Frauenheilkunde |  | Neurochirurgie |
|  | Gastroenterologie |  | Neurologie |
|  | Gefäßchirurgie |  | Neuropädiatrie |
|  | Geriatrie |  | Neuropathologie |
|  | Geschlechtskrankheiten und Dermatologie |  | Orthopädie und Unfallchirurgie |
|  | Gynäkologische Endokrinologie und Reproduktionsmedizin |  | Physikalische und Rehabilitative Medizin |
|  | Gynäkologische Onkologie |  | Plastische Chirurgie |
|  | Hals-Nasen-Ohrenheilkunde |  | Pneumologie |
|  | Hämatologie und Onkologie |  | Psychiatrie und Psychotherapie |
|  | Humangenetik |  | Psychologischer Psychotherapeut |
|  | Infektiologie |  | Psychotherapeutisch tätiger Arzt |
|  | Innere Medizin/  Rheumatologie |  | Thoraxchirurgie |
|  | Kardiologie |  | Transfusionsmedizin |
|  | Kinder- und Jugendlichen-Psychotherapeut |  | Urologie |
|  | Kinder- und Jugendmedizin |  | Viszeralchirurgie |
|  | Kinder- und Jugendpsychiatrie und -psychotherapie |  | praktischer Arzt |
|  | Sonstige:  [Bitte im Feld rechts eintragen.] |  | |

1. **Sind Sie in der psychotherapeutischen Versorgung tätig?**

|  | ja | 🡪 weiter mit **Frage 18** |
| --- | --- | --- |
|  | nein | 🡪 weiter mit **Frage 19** |

1. **Bitte geben Sie an, für welche psychotherapeutische Verfahren Sie eine fachliche Befähigung haben.**

[Mehrfachantworten sind möglich.]

|  | Analytische Psychotherapie |
| --- | --- |
|  | Tiefenpsychologisch fundierte Psychotherapie |
|  | Verhaltenstherapie |
|  | Systemische Therapie |

1. **Sie haben nun einige Fragen zur Videosprechstunde beantwortet. Können Sie sich grundsätzlich vorstellen, Videosprechstunden anzubieten?**

|  | ja |
| --- | --- |
|  | nein |

**Vielen Dank für Ihre Teilnahme!**
