# Supplementary material for: Attitudes Toward Video Consultations From the Perspective of Physicians and Psychotherapists in German Outpatient Care After the COVID-19 Pandemic: Survey Study
Source: J Med Internet Res. 2026 Jan 6;28:e73757. doi: 10.2196/73757 (PMC12774393; doi:10.2196/73757)
Supplement: Multimedia Appendix 2 [file jmir-v28-e73757-s002.docx]

## **Appendix 2: Subgroup-analysis for provision of VC.**

|  | | **Yes n/N (%)** | **No n/N (%)** | **χ²^a^** | **P value** | **effect size^a^** |
| --- | --- | --- | --- | --- | --- | --- |
| **gender n=5471** | | | | 234.987 | <.001 | .207 |
|  | Female | 1457/3109 (46.9) | 1652/3109 (53.1) |  |  |  |
|  | Male | 627/2362 (26.5) | 1735/2362 (73.5) |  |  |  |
| **age group n=5490** | | | | 59.314 | <.001 | .104 |
|  | < 40 years | 374/743 (50.3) | 369/743 (49.7) |  |  |  |
|  | 40 – 50 years | 564/1470 (38.4) | 906/1470 (61.6) |  |  |  |
|  | 51 – 60 years | 687/1932 (35.6) | 1.245/1932 (64.4) |  |  |  |
|  | > 60 years | 466/1345 (34.6) | 879/1345 (65.4) |  |  |  |
| **community size of practice location n=5477** | | |  | 131.924 | <.001 | .155 |
|  | rural community | 116/367 (31.6) | 251/367 (68.4) |  |  |  |
|  | small town | 285/954 (29.9) | 669/954 (70.1) |  |  |  |
|  | middle town | 430/1402 (30.7) | 972/1402 (69.3) |  |  |  |
|  | large city | 1.255/2754 (45.6) | 1.499/2754 (54.4) |  |  |  |
| **type of practice *^b^* n=5304** | | | | 292.140 | <.001 | .235 |
|  | individual practice | 1159/2705 (42.8) | 1546/2705 (57.2) |  |  |  |
|  | joint practice | 346/1427 (24.2) | 1081/1427 (75.8) |  |  |  |
|  | group practice | 399/700 (57) | 301/700 (43) |  |  |  |
|  | medical care unit | 110/472 (23.3) | 362/472 (76.7) |  |  |  |
| **ownership of practice n=5304** | | |  | 154,658 | <.001 | .171 |
|  | self-employed | 1779/4218 (42.2) | 2439/4218 (57.8) |  |  |  |
|  | employed | 235/1086 (21.6) | 851/1086 (78.4) |  |  |  |
| **area of medical care n=5482** | | |  | 1840.293 | <.001 | .579 |
|  | primary care | 308/1572 (19.6) | 1264/1572 (80.4) |  |  |  |
|  | specialist care | 203/1736 (11.7) | 1533/1736 (88.3) |  |  |  |
|  | psychotherapeutic care | 1579/2174 (72.6) | 595/2174 (27.4) |  |  |  |

***^a^ chi square test with Cramer’s-V effect size***

***^b^ German Terms: joint practice = Berufsausübungsgemeinschaft; group practice = Praxisgemeinschaft; medical care unit = Medizinisches Versorgungszentrum***
