# Supplementary material for: Attitudes Toward Video Consultations From the Perspective of Physicians and Psychotherapists in German Outpatient Care After the COVID-19 Pandemic: Survey Study
Source: J Med Internet Res. 2026 Jan 6;28:e73757. doi: 10.2196/73757 (PMC12774393; doi:10.2196/73757)
Supplement: Multimedia Appendix 3 [file jmir-v28-e73757-s003.docx]

**Appendix 3: Subgroup-analysis for interest in VC provision.**

|  | | **Yes n/N (%)** | **No n/N (%)** | **χ²^a^** | **P value** | **effect size^a^** |
| --- | --- | --- | --- | --- | --- | --- |
| **gender n=5400** | | | | 101.387 | <.001 | .137 |
|  | Female | 2600/3070 (84.7) | 470/3070 (15.3) |  |  |  |
|  | Male | 1715/2330 (73.6) | 615/2330 (26.4) |  |  |  |
| **age group n=5420** | | | | 175.303 | <.001 | .18 |
|  | < 40 years | 689/743 (92.7) | 54/743 (7.3) |  |  |  |
|  | 40 – 50 years | 1229/1445 (85.1) | 216/1445 (14.9) |  |  |  |
|  | 51 – 60 years | 1476/1910 (77.3) | 434/1910 (22.7) |  |  |  |
|  | > 60 years | 937/1322 (70.9) | 385/1322 (29.1) |  |  |  |
| **community size of practice localtion n=5412** | | |  | 40.404 | <.001 | .086 |
|  | rural community | 279/265 (76.4) | 86/365 23.6) |  |  |  |
|  | small town | 710/946 (75.1) | 236/946 (24.9) |  |  |  |
|  | middle town | 1070/1381 (77.5) | 311/1381 (22.5) |  |  |  |
|  | large city | 2264/2720 (83.2) | 456/2720 (16.8) |  |  |  |
| **area of medical care n=5423** | | |  | 313.466 | <.001 | .240 |
|  | primary care | 1128/1547 (72.9) | 419/1547 (27.1) |  |  |  |
|  | specialist care | 1227/1719 (71.4) | 492/1719 (28.6) |  |  |  |
|  | psychotherapeutic care | 1979/2157 (91.7) | 178/2157 (8.3) |  |  |  |
| **VC experience n=5395** | | | | 768,624 | <.001 | .377 |
|  | yes | 2043/2060 (99.2) | 17/2060 (0.8) |  |  |  |
|  | no | 2270/3335 (68.1) | 1065/3335(31.9) |  |  |  |

***^a^ chi square test with Cramer’s-V effect size***
