# Supplementary material for: Attitudes Toward Video Consultations From the Perspective of Physicians and Psychotherapists in German Outpatient Care After the COVID-19 Pandemic: Survey Study
Source: J Med Internet Res. 2026 Jan 6;28:e73757. doi: 10.2196/73757 (PMC12774393; doi:10.2196/73757)
Supplement: Multimedia Appendix 4 [file jmir-v28-e73757-s004.docx]

## **Appendix 4: Attitudes on suitable medical fields (only for participants with VC experience).**

|  | highly unsuitable | unsuitable | to some extent suitable | suitable | very suitable |
| --- | --- | --- | --- | --- | --- |
|  | **n, %** | **n, %** | **n, %** | **n, %** | **n, %** |
| **Mental and behavioral disorders** | | | | | |
| **Non-organic sleep disorders** | 26, 1.3 | 102, 5.1 | 571, 28.6 | 814, 40.8 | 482, 24.2 |
| **Anxiety disorders** | 42, 2.1 | 176, 8.7 | 686, 34.1 | 698, 34.7 | 412, 20.5 |
| **Affective disorders** | 39, 2.0 | 163, 8.2 | 666, 33.5 | 714, 35.9 | 409, 20.5 |
| **Obsessive-compulsive disorders** | 43, 2.2 | 224, 11.3 | 755, 38.2 | 602, 30.5 | 350, 17.7 |
| **Sexual dysfunctions** | 49, 2.7 | 204, 11.1 | 641, 34.9 | 606, 33.0 | 336, 18.3 |
| **Eating disorders** | 57, 2.9 | 280, 14.4 | 776, 40.0 | 549, 28.3 | 278, 14.3 |
| **Reactions to severe stress and adjustment disorders** | 85, 4.2 | 293, 14.5 | 723, 35.7 | 568, 28.1 | 354, 17.5 |
| **Somatoform disorders and dissociative disorders (conversion disorders)** | 83, 4.1 | 350, 17.5 | 783, 39.1 | 520, 26.0 | 265, 13.2 |
| **Personality and behavioral disorders** | 108, 5.5 | 361, 18.3 | 801, 40.6 | 451, 22.9 | 251, 12.7 |
| **Behavioral and emotional disorders with onset in childhood and adolescence** | 123, 7.8 | 371, 23.5 | 586, 37.1 | 322, 20.4 | 177, 11.2 |
| **Schizophrenia** | 340, 19.3 | 707, 40.1 | 522, 29.6 | 134, 7.6 | 61, 3.5 |
| **Delusional disorders caused by psychotropic substances** | 240, 13.1 | 584, 31.8 | 652, 35.5 | 234, 12.7 | 127, 6.9 |
| **Chronic conditions** | | | | | |
| **Chronic pain, e. g. headaches** | 18, 1.2 | 95, 6.3 | 468, 30.9 | 635, 41.9 | 301, 19.8 |
| **Metabolic disorders, e. g. chronic diabetes mellitus** | 24, 2.4 | 121, 12.0 | 400, 39.6 | 333, 33.0 | 131, 13.0 |
| **Chronic allergies** | 40, 3.9 | 162, 15.9 | 405, 39.7 | 299, 29.3 | 114, 11.2 |
| **Dermatological diseases, e. g. chronic psoriasis** | 52, 5.3 | 196, 19.8 | 373, 37.7 | 250, 25.3 | 118, 11.9 |
| **Cardiovascular diseases, e. g. arterial hypertension, coronary heart disease** | 48, 4.8 | 257, 25.5 | 402, 39.8 | 224, 22.2 | 78, 7.7 |
| **Musculoskeletal disorders, e. g. chronic back pain** | 75, 7.1 | 267, 25.3 | 427, 40.5 | 200, 19.0 | 86, 8.2 |
| **Neurological diseases, e. g. chronic post-stroke condition** | 98, 9.6 | 272, 26.5 | 412, 40.2 | 172, 16.8 | 71, 6.9 |
| **ENT (ear, nose, and throat) diseases, e. g. chronic rhinosinusitis** | 57, 6.5 | 259, 29.6 | 365, 41.7 | 142, 16.2 | 53, 6.1 |
| **Pulmonary diseases, e. g. chronic bronchial asthma, chronic obstructive pulmonary disease** | 59, 6.1 | 293, 30.3 | 402, 41.5 | 151, 15.6 | 63, 6.5 |
| **Vascular diseases, e. g. chronic peripheral arterial disease** | 62, 6.9 | 331, 36.6 | 347, 38.4 | 113, 12.5 | 51, 5.6 |
| **Ophthalmic diseases, e. g. chronic cataract** | 118, 15.1 | 324, 41.3 | 243, 31.0 | 61, 7.8 | 38, 4.8 |
| **Gastrointestinal diseases, e. g. chronic inflammatory bowel diseases** | 31, 3.0 | 182, 17.8 | 457, 44.8 | 248, 24.3 | 103, 10.1 |
| **Neoplastic diseases** | 128, 12.7 | 230, 22.8 | 419, 41.5 | 156, 15.5 | 76, 7.5 |
| **Acute illnesses** | | | | | |
| **Acute headaches** | 67, 5.4 | 171, 13.8 | 481, 38.9 | 369, 29.8 | 150, 12.1 |
| **Acute dermatoses** | 104, 11.3 | 246, 26.7 | 337, 36.6 | 174, 18.9 | 61, 6.6 |
| **Acute gastrointestinal infections** | 119, 12.3 | 246, 25.4 | 387, 40.0 | 166, 17.1 | 50, 5.2 |
| **Acute musculoskeletal pain** | 80, 7.6 | 282, 27.0 | 460, 44.0 | 172, 16.4 | 52, 5.0 |
| **Acute skin injuries** | 154, 15.8 | 330, 33.9 | 298, 30.6 | 144, 14.8 | 48, 4.9 |
| **Acute ocular inflammations** | 186, 19.8 | 340, 36.2 | 245, 26.1 | 128, 13.6 | 41, 4.4 |
| **Acute respiratory infections** | 151, 15.5 | 325, 33.4 | 324, 33.3 | 132, 13.6 | 40, 4.1 |
| **Acute urinary tract infections** | 162, 16.9 | 383, 40.0 | 292, 30.5 | 88, 9.2 | 32, 3.3 |
| **Acute sexually transmitted infections** | 216, 23.7 | 373, 41.0 | 227, 24.9 | 68, 7.5 | 26, 2.9 |
| **Acute ear inflammations** | 227, 24.2 | 474, 50.5 | 182, 19.4 | 36, 3.8 | 20, 2.1 |
