# Supplementary material for: Attitudes Toward Video Consultations From the Perspective of Physicians and Psychotherapists in German Outpatient Care After the COVID-19 Pandemic: Survey Study
Source: J Med Internet Res. 2026 Jan 6;28:e73757. doi: 10.2196/73757 (PMC12774393; doi:10.2196/73757)
Supplement: Multimedia Appendix 5 [file jmir-v28-e73757-s005.docx]

|  | VC experience ^a^ | |
| --- | --- | --- |
|  | **P value** | **effect size ^a^** |
| Taking a patient’s medical history | <.001 | .125 |
| (Further) Diagnostic work-up | <.001 | .428 |
| Therapy treatment planning | <.001 | .288 |
| Issuing prescriptions for drugs and remedies | <.001 | .197 |
| Issuing incapacity certificate | <.001 | .205 |
| Discussion of test results | <.001 | .166 |
| Follow-up checks (e.g. wound healing medication) | <.001 | .365 |
| Individual psychiatric/ psychotherapeutic consultations | <.001 | .453 |
| Group sessions (e.g. in psycho-therapy) | <.001 | .173 |

## **Appendix 5: Association and effect size of suitable types of treatment for participants with VC experience.**

***^a^ chi square test with Cramer’s-V effect size***
