# Supplementary material for: Attitudes Toward Video Consultations From the Perspective of Physicians and Psychotherapists in German Outpatient Care After the COVID-19 Pandemic: Survey Study
Source: J Med Internet Res. 2026 Jan 6;28:e73757. doi: 10.2196/73757 (PMC12774393; doi:10.2196/73757)
Supplement: Multimedia Appendix 6 [file jmir-v28-e73757-s006.docx]

## **Appendix 6: Association/Correlation and effect size of suitable types of treatment (only for participants with VC experience).**

|  | gender^a^ | | age groups^b^ | | community size of practice location^b^ | | area of medical care^a^ | | ownership of practice^a^ | | type of practice^a^ | |
| --- | --- | --- | --- | --- | --- | --- | --- | --- | --- | --- | --- | --- |
|  | **P value** | **effect size** | **P value** | **effect size** | **P value** | **effect size** | **P value** | **effect size** | **P value** | **effect size** | **P value** | **effect size** |
| Taking a patient’s medical history | .007 | .083 | <.001 | -.094 | <.001 | -.071 | <.001 | .229 | <.001 | .108 | <.001 | .094 |
| (Further) Diagnostic work-up | n.s. |  | <.001 | -.087 | n.s. |  | <.001 | .145 | n.s. |  | <.001 | .077 |
| Therapy treatment planning | n.s. |  | <.001 | -.094 | .016 | -.039 | .05 | .062 | .039 | .072 | n.s. |  |
| Issuing prescriptions for drugs and remedies | n.s |  | n.s. |  | n.s. |  | n.s. |  | n.s. |  | n.s. |  |
| Issuing incapacity certificate | n.s. |  | n.s. |  | n.s. |  | <.001 | .135 | .006 | .122 | n.s. |  |
| Discussion of test results | <.001 | .150 | n.s. |  | n.s. |  | <.001 | .213 | n.s. |  | <.001 | .108 |
| Follow-up checks (e.g. wound healing medication) | n.s. |  | n.s. |  | n.s. |  | <.001 | .167 | .002 | .116 | .002 | .091 |
| Individual psychiatric/ psychotherapeutic consul-tations | <.001 | .125 | .031 | -.040 | n.s. |  | <.001 | .182 | .057 | .070 | <.001 | .088 |
| Group sessions (e.g. in psycho-therapy) | n.s. |  | n.s. |  | .035 | .052 | n.s. |  | n.s. |  | n.s. |  |

***^a^chi square test with Cramer’s-V effect size***

***^b^Kendall's-Tau-c***

***The level of statistical significance is set at α = .05 (p ≤ .05)***
